# Supplementary material for: GC-MS Silylation Derivative Method to Characterise Black BIC® Ballpoint 2-Phenoxyethanol Ratio Evaporation Profile—A Contribution to Ink Ageing Estimation
Source: Molecules. 2023 Jun 15;28(12):4781. doi: 10.3390/molecules28124781 (PMC10302093; doi:10.3390/molecules28124781)
Supplement: Supplementary file 1 [file molecules-28-04781-s001.zip › molecules-2254643-supplementary.pdf]

Supplementary Materials

GC-MS Silylation Derivative Method to Characterise Black BIC® Ballpoint 2-Phenoxyethanol Ratio Evaporation Profile—A Contribution to Ink Ageing Estimation

Teresa Argente Leal [1], Carla Ferreira [1,2], Ana Ribeiro [1], Samir Marcos Ahmad [1,2], Alexandre Quintas [1,2,*] and Alexandra Bernardo [1,2,*]

[1] Forensic and Psychological Sciences Laboratory Egas Moniz, Campus Universitário—Quinta da Granja, Monte da Caparica, 2829-511 Caparica, Portugal

[2] Molecular Pathology and Forensic Biochemistry Laboratory, Centro de Investigação Interdisciplinar Egas Moniz (CiiEM), Instituto Universitário Egas Moniz (IUEM), Campus Universitário—Quinta da Granja, Monte da Caparica, 2829-511 Caparica, Portugal

* Correspondence: aquintas@egasmoniz.edu.pt (A.Q.); abernardo@egasmoniz.edu.pt (A.B.)

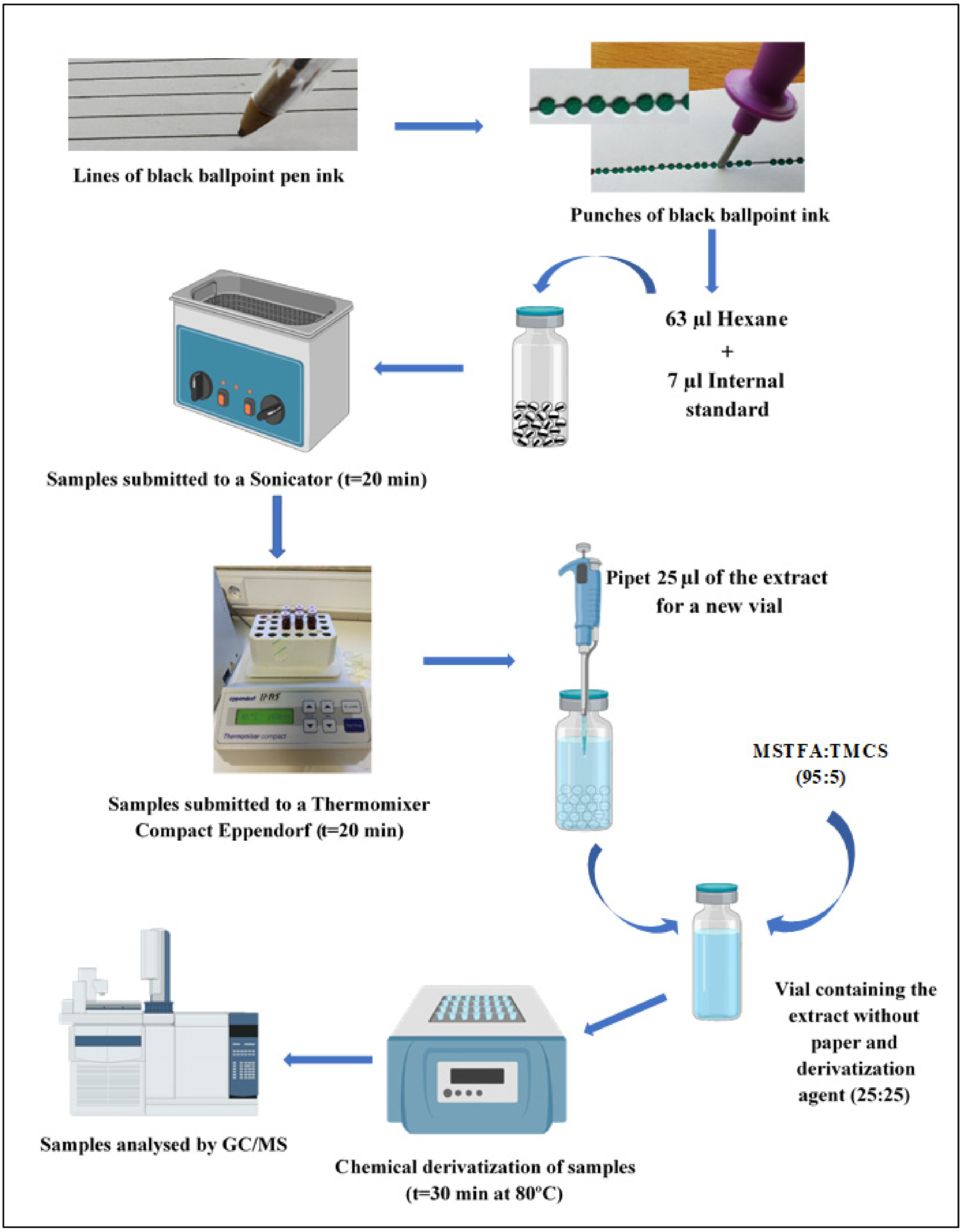

Figure S1 - Extraction procedure for each ink entry. This procedure was performed for each sample in triplicate.
